# Study of the Interaction of Trastuzumab and SKOV3 Epithelial Cancer Cells Using a Quartz Crystal Microbalance Sensor

**DOI:** 10.3390/s150305884

**Published:** 2015-03-10

**Authors:** Louise Elmlund, Camilla Käck, Teodor Aastrup, Ian A. Nicholls

**Affiliations:** 1 Bioorganic & Biophysical Chemistry Laboratory, Linnæus University Centre for Biomaterials Chemistry, Department of Chemistry & Biomedical Sciences, Linnæus University, SE-39182 Kalmar, Sweden; E-Mail: louise.elmlund@lnu.se; 2 Attana AB, Björnnäsvägen 21, SE-11419 Stockholm, Sweden; E-Mails: camilla.kack@attana.com (C.K.); teodor.aastrup@attana.com (T.A.); 3 Department of Chemistry–BMC, Uppsala University, Box 576, SE-75123 Uppsala, Sweden

**Keywords:** quartz crystal microbalance, breast cancer, cell-based biosensor, Herceptin, trastuzumab, HER2

## Abstract

Analytical methods founded upon whole cell-based assays are of importance in early stage drug development and in fundamental studies of biomolecular recognition. Here we have studied the binding of the monoclonal antibody trastuzumab to human epidermal growth factor receptor 2 (HER2) on human ovary adenocarcinoma epithelial cancer cells (SKOV3) using quartz crystal microbalance (QCM) technology. An optimized procedure for immobilizing the cells on the chip surface was established with respect to fixation procedure and seeding density. Trastuzumab binding to the cell decorated sensor surface was studied, revealing a mean dissociation constant, *K*_D_, value of 7 ± 1 nM (standard error of the mean). This study provides a new perspective on the affinity of the antibody-receptor complex presented a more natural context compared to purified receptors. These results demonstrate the potential for using whole cell-based QCM assay in drug development, the screening of HER2 selective antibody-based drug candidates, and for the study of biomolecular recognition. This real time, label free approach for studying interactions with target receptors present in their natural environment afforded sensitive and detailed kinetic information about the binding of the analyte to the target.

## 1. Introduction

Cell-based assays are interesting for drug development and diagnostics. Even though the standard methods for early drug development are based on *in vitro*-assays, cell-based assays offer extended possibilities for understanding the biology including *in vivo*-effects [1,2]. Traditional methods for binding studies between cell membrane-incorporated receptors and corresponding antibodies are often based on purified receptors or cell lysate. Immunoassays, e.g., ELISA, can be time consuming, expensive and for some assays, also not sufficiently sensitive [3]. Moreover, fluorescence labeling of antibodies to intact cells (immunocytochemistry) and flow cytometry have limitations in quantification and qualification of the binding, and for cell-based systems in general there is a need to develop protocols that take into account the stability of the cells. Additionally, labeling can affect the interaction properties and increase the non-specific binding [4], which highlights the fundamental benefits offered by label-free analytical tools. The affinity between drug and target, including on- and off-rates, are evaluated for drug development [5,6] which increases the need of a tool for proper affinity data in its natural environment. Biosensors can offer a time efficient, label-free assay for studying binding with affinity features in real time. Limitations of traditional biosensors are the need for isolated and purified target molecules immobilized on the sensor surface where the target is not present in its natural context, possibly resulting in undesirable conformation alterations of the target molecule [7]. To overcome that limitation and offer a target binding assay in a more biological context, cell-based biosensors have been explored and shown to be promising tools [8,9,10,11,12]. Here we have addressed the use of the human ovary adenocarcinoma epithelial cancer (SKOV3) cell line, with an overexpression of the breast cancer-related human epidermal growth factor receptor 2 (HER2), as a means for studying the interaction with the monoclonal antibody-based drug trastuzumab, and with the long term goal of establishing methods for the rapid screening of new antibody-based candidate drugs.

The quartz crystal microbalance (QCM) is a piezoelectricity-based biosensor technology for quantitative and qualitative measurements of binding affinity. Due to its robustness, low-cost and ease-of-use it has become a powerful label-free tool to identify binding in real time [9,13,14,15,16,17] suitable for a wide range of molecules, from small organic compounds to large proteins. Like most biosensors, the QCM technique has traditionally been used mostly for studying the interaction between purified antibodies and antigens or related substances. The first immunosensor based on piezoelectric detection was reported in 1972 by Shons and co-workers [18,19,20]. The QCM technique has since been undergone significant development, whereby today it is possible to use synthetic polymer-based antibody mimics [21,22] and even cells as sensor recognition elements [7,23,24,25,26,27,28].

The initial development of applications based upon cells attached to QCM surfaces have included the monitoring of cell adhesion [9], the effects of anti-cancer treatments on cells [29,30], the detection of cancer cells [31,32] and the affinity of antibodies for a cell membrane receptor [33]. The possibility of studying the binding of an analyte to cells attached to resonator surfaces allows for the interaction to be examined in a more natural environment, *i.e.*, together with other cell membrane components that may affect the binding properties [7,23,24,25].

**Figure 1:**
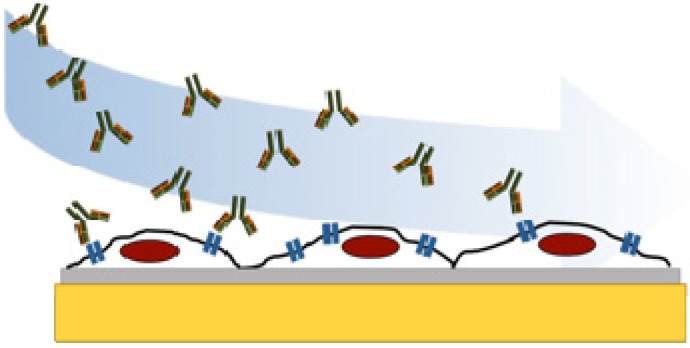
Schematic illustration of trastuzumab (green/yellow) binding to HER2 receptor (blue) on SKOV3 cells attached to QCM cell chip.

In this work we have studied the binding of the monoclonal antibody trastuzumab (commercially known as Herceptin™) to the receptor HER2 on SKOV3 epithelial cancer cells using quartz crystal microbalance studies (Figure 1). Overexpressed HER2 are found in aggressive forms of breast cancer and are therefore considered an important target for diagnosis and treatment [34]. The humanized anti-HER2 antibody trastuzumab is widely used for clinical diagnosis and treatment of these cancer forms since it has found to bind to the HER2 and induce apoptosis. We have, in this study, developed a system for measuring the interaction to HER2 on SKOV3 epithelial cancer cells attached to a COP-1 QCM chip. The HER2 is overexpressed in this cell line. Since cell adhesion is cell dependent [9,35] and may affect the measured frequency shift [36] it was important to study the impact of preparation procedure in order to acquire accurate binding data. The SKOV3 cell line was attached and fixed to COP-1 chips and trastuzumab was passed over the surface allowing binding to the HER2 on the cell membrane.

## 2. Experimental Section

### 2.1. Chemicals

Foetal Bovine Serum (FBS, heat inactivated), RPMI 1640 medium (1X, GIBCO^®^ by Life Technologies^TM^, Grand Island, NY, USA), penicillin-streptomycin (liquid, GIBCO^®^), 4',6-diamidino-2-phenylindole (DAPI) Nucleic Acid Stain (Invitrogen Molecular Probes by Life Technologies^TM^), 1× PBS pH 7.4 and 10× PBS pH 7.2 were purchased from Life Technologies (Paisley, UK). Formaldehyde (37%), glutaraldehyde (25%), glycine and 0.25% trypsin-EDTA (1×, GIBCO^®^) were from Sigma-Aldrich (St Louis, MO, USA). Herceptin^TM^ (trastuzumab) was from Roche (Welwyn Garden City, UK). The water used was purified using Milli-Q system (Millipore AB, Billerica, MA, USA). SKOV3 cells were received from ATCC (Manassas, VA, USA). COP-1 (cell optimized polystyrene) sensor chips were purchased from Attana AB (Stockholm, Sweden).

### 2.2. Preparation of Cell Chips

Human ovary adenocarcinoma cells (SKOV3) were cultured in RPMI 1640 (1×) medium, complemented with 10% FBS and 1% penicillin-streptomycin. Cells were cultured in T flasks in a humidified incubator with 5% CO_2_/95% air atmosphere at 37 °C. Before adhesion of cells on sensor chip the cells were washed in PBS and trypsinized with 0.25% trypsin-EDTA mixture for 5–10 min at 37 °C. After addition of at least an equal amount of media the cell suspension was transferred into a falcon tube and centrifuged at 1000 rpm for 5 min to obtain a pellet. The pellet was resuspended in an appropriate amount of media and mixed carefully to obtain a homogenous suspension. Cell chips were prepared by incubating aliquots of cell suspension on COP-1 chip in a humidified incubator. After 24 h of incubation, the cell suspension was gently removed and the chips were washed with cold PBS. The cells were then fixed using either formaldehyde in PBS or glutaraldehyde in water at 4 °C for 10 min followed by soaking with PBS (3 × 5 min). Finally, the chips were stained by addition of 50 μL of DAPI (0.6 nM in PBS) to each chip, incubation for 4 min at room temperature in the dark followed by rinsing in PBS. Cell immobilization was confirmed using an epifluorescence microscope (Eclipse E400, Nikon, Tokyo, Japan) equipped with an appropriate DAPI filter and a digital camera (Nikon DS-U1). The cell chips were stored in PBS at 4 °C in the dark until use.

### 2.3. Quartz Crystal Microbalance Studies

QCM studies were conducted under flow injection analysis (FIA) conditions using an automated Attana Cell 200 instrument (Attana AB, Stockholm, Sweden). Typically, a SKOV3 coated chip was placed in a chip holder with a chamber volume of 1.46 μL and docked in the instrument. The temperature was set to 22 °C and the flow rate to 20 μL/min. Running buffer, PBS (pH 7.4), was passed over the chip until stabilization of baseline (frequency change ≤0.5 Hz over 600 s). The binding of trastuzumab was studied by 35 μL injections (105 s) followed by dissociation in PBS for 300 s. For removal of any remaining analyte, 12.5 μL glycine (10 mM in water, pH 2) was injected (38 s) followed by continuous PBS flow until stabilization of baseline before next injection. Data was collected using and analyzed with Attester software (Attana AB).

## 3. Results and Discussion

SKOV3 cancer cell line was cultivated in T flasks until 80% of confluence, moved to suspension and cultivated for 24 h on top of COP-1 chip, optimized for cell attachment. To ensure accurate binding data, the preparation procedure was optimized before using the COP-1 chips as sensor resonators. Trastuzumab was passed over the cell chip allowing the antibody to bind with its target receptor, HER2, present in the SKOV3 cell membrane.

### 3.1. Fixation of SKOV3 Cells to COP-1 Chips

**Figure 2:**
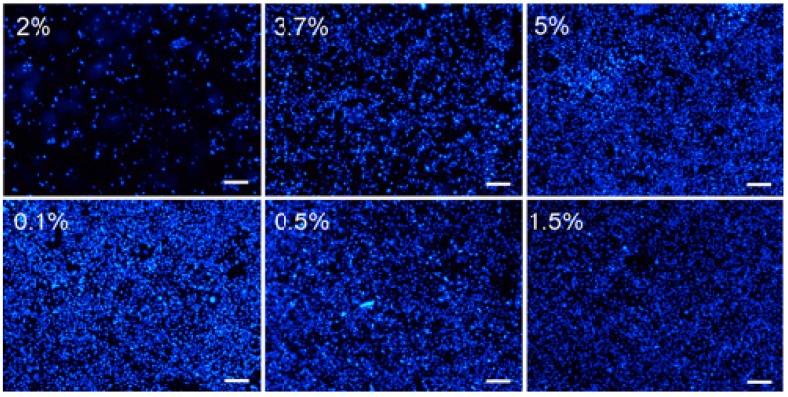
Fluorescence micrographs of DAPI stained SKOV3 cells on QCM cell chips fixed using FA (top row) or GA (bottom row). Scale bars 200 μm.

There is no doubt that good fixation should preserve the cells without affecting their components [37]. It is crucial however that when studying interactions to whole cells that the membrane proteins stay intact without changing epitopes. The outcome is dependent upon several factors such as fixation agent, concentration and exposure time. The most common fixation strategies use aldehydes, which form cross-linkages between proteins to preserve the cells. The binding properties of the SKOV3 cell chips were studied after fixation using two of the most widely used aldehydes, formaldehyde (FA) and glutaraldehyde (GA), in different concentrations. After incubating chips in cell suspension (corresponding to 4 × 10^4^ cells per sensor surface), the cells were fixed with aldehyde followed by staining using DAPI to visualize the nuclei of the cells attached to the sensor surface. As shown in Figure 2, cells treated with FA exhibited increased densities on the chips with higher concentrations of aldehyde. GA, on the other hand, showed similar or even less coverage at higher concentrations. Preliminary binding studies were performed using two chips, one fixed with 3.7% FA and one with 0.5% GA. 

**Figure 3:**
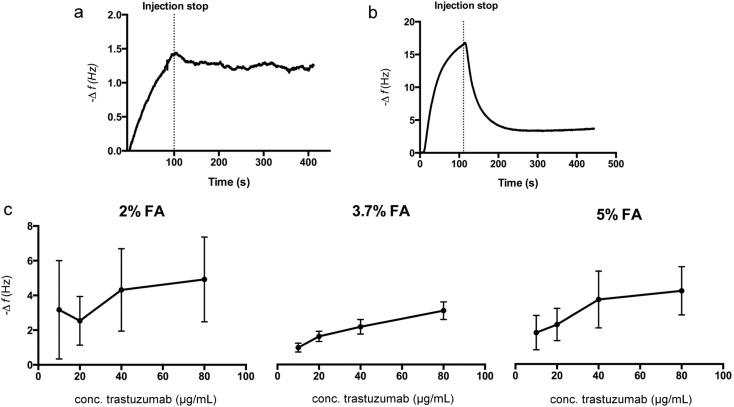
Representative sensorgrams after injection of 20 μg/mL trastuzumab to chips fixed using (**a**) 3.7% FA and (**b**) 0.5% GA; (**c**) Maximum frequency response after injection of different concentrations of trastuzumab to FA fixed cell chips. Error bars represent standard deviation (SD) for triplicate injections on three chips.

The chip treated with 3.7% FA showed a binding curve with both association and dissociation evident, indicating specific binding of trastuzumab to the cells attached to the chip (Figure 3a). On the other hand, the maximum frequency shift (−Δf) was significantly higher for the 0.5% GA fixed cell chip and the profile of the binding curve showed a strong and rapid decrease in frequency during injection, followed by a fast return to baseline after injection (Figure 3b). This observation indicated a greater degree of weak or non-specific binding to this chip, as reflected in the immediate commencement of return to baseline after completion of injection non-specific binding. GA is widely used because of its efficiency in crosslinking but the effect is also cell dependent and can cause cell damage even if a low concentration is used [38]. There are also known problems with residual unreacted aldehyde functionalities, which can contribute to non-specific binding of antibodies, thus giving a false positive result [39]. Since the GA fixed cell chip showed high cell coverage, the non-specific binding could result from a negatively affected HER2, binding to free aldehydes or even cell lysis due to the impact of the fixation solution. Following these preliminary studies a more extensive study of trastuzumab (10–80 μg/mL) binding to the FA fixed cell chips was conducted (Figure 3c). For chips fixed using 3.7% and 5% FA, the maximum frequency response indicated a concentration dependent binding to the cell chips. For the 2% FA fixed cell chips the maximum frequency response did not follow the same trend, moreover, the response showed a much wider distribution between different chips (*n =* 3). This could indicate non-specific binding, which supports the results from the DAPI staining where this chip showed a lower cell density on the chip surface. Additionally, the cell chips fixed with 2% FA showed a different binding curve profile than those fixed with 3.7% FA, with a rapid frequency increase during injection and rapid decrease after injection stop, indicating non-specific binding (not shown). The 5% FA fixed cell chips also showed a slightly wider distribution of maximum frequency responses (Figure 3c) as compared to the chip fixed with 3.7% FA. Considering the high cell density on these chips, which also indicates non-specific binding, this could be caused by a toxic effect from this high concentration of FA.

### 3.2. Seeding Density

**Figure 4:**
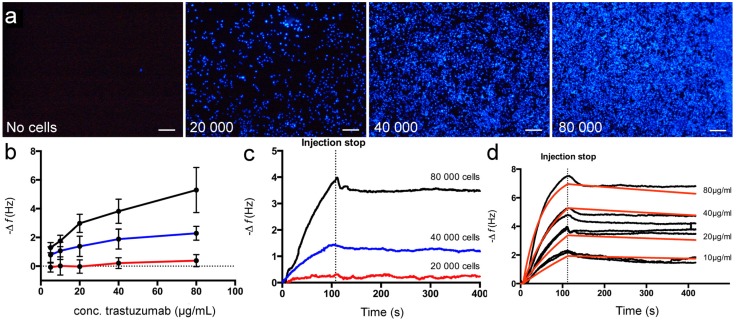
(**a**) Fluorescence micrographs of DAPI stained SKOV3 cells on COP-1 chips prepared using increasing seeding density. Scale bars 200 μm; (**b**) Maximum frequency response for different concentrations of trastuzumab to 2 × 10^4^ (red), 4 × 10^4^ (blue) and 8 × 10^4^ (black) cells per chip. Error bars represent SD for triplicate injections on three different chips; Representative sensorgrams after injection of (**c**) 20 μg/mL trastuzumab to chips prepared using increasing seeding density and (**d**) trastuzumab binding (black curves) to cell chip seeded with corresponding to 8 × 10^4^ cells/surface. Red curves represent theoretical curve fitting using a mass transport limited model. The mean *K*_D_ value was calculated to 7 ± 1 nM (standard error of the mean). Data from two cell chips, with four analyte concentrations.

To examine how seeding density affects the binding capacity of trastuzumab, COP-1 chips were incubated in cell suspensions with different concentrations corresponding to seeding densities of 2 × 10^4^, 4 × 10^4^ and 8 × 10^4^ cells per sensor surface (15.9 mm^2^). After incubation (24 h), the chips were fixed with 3.7% formaldehyde, stained and examined using fluorescence microscopy (Figure 4a). All chips showed well-distributed cells with no indication of cell cluster growth. As expected, cell density increased if more cells were added. Next, the binding properties of the chips were examined using trastuzumab injections (5–80 μg/mL) under FIA conditions (Figure 4b). The maximum frequency response increased with increasing seeding density for all trastuzumab concentrations. For the lowest seeding density, 2 × 10^4^ cells/chip, very low binding with no concentration dependence was observed, most likely due to the low surface coverage (Figure 4a). For the higher seeding densities, the maximum frequency responses were higher and concentration dependent, reflecting the higher surface coverage of these chips. To verify that the frequency shifts resulted from the specific binding of trastuzumab, the sensorgrams were examined in more detail (Figure 4c) using 20 μg/mL injections. The binding curves showed association and dissociation curvature for all seeding densities. The injection part of the curves for the cell chips seeded with corresponding to 2 × 10^4^ showed saturation and 4 × 10^4^ cells/surface approached saturation, reaching its maximum frequency response quickly, while for chips seeded with 8 × 10^4^ cells/surface showed no saturation using this trastuzumab concentration.

### 3.3. Kinetic Evaluation of Trastuzumab Binding to SKOV3 Cell Chips

In order to determine the affinity for trastuzumab binding to the SKOV3 cell chip, the dissociation constant (K_D_) was calculated using binding data from two prepared chips using a seeding density of 8 × 10^4^ cells/chip and fixed using 3.7% FA (Figure 4d). Sensorgrams from different concentrations of the analyte were fitted to a mass transport limited model. The diffusion rate of the analyte, which is carried form the bulk solution to the sensor surface needs to be considered. Mass transport limitations occur when the association rate is fast and the diffusion of the analyte becomes limiting for the interaction [6,40]. Mass transport limitations can be recognized in the sensorgram by a linear appearance in the early stages of the association phase and non-exponential dissociation phase. Such effects can be avoided by using lower surface densities, by increasing the flow rate during analysis, or by using a calculation model for mass transport limitation, as here. K_D_ values were calculated resulting in a mean K_D_ value of 7 ± 1 nM (standard error of the mean). This value is strongly correlated to other published data for the interaction using other cell-based assays (K_D_ = 5 nM according to the manufacturer of Herceptin^TM^). Other reported studies of the kinetics of this interaction, Carter *et al.* and Bostrom *et al.* have reported dissociation constants of 0.1 nM [41] and 0.5 nM [42] for trastuzumab binding to immobilized HER2 (extra cellular domain) using ELISA and SPR technology, respectively. Accordingly, these studies of antibody-HER2 interactions provide a new perspective on the affinity in a more natural environment. Moreover, this study demonstrates the potential for using whole cell-based QCM studies in drug development and for the study of biomolecular recognition.

Clinical tests for diagnostics are often time-consuming and not sufficiently sensitive, resulting in false-positive or negative outcomes. Improving assay reliability while retaining high sensitivity is therefore of great importance, in particular for the early stage detection of diseases such as various forms of cancer. Furthermore, for drug development; speed, quality and cost are in focus and time-effective high throughput tools are important [5]. Antibody-mediated diagnostics and treatments have proven of value in the diagnosis and treatment of several diseases, e.g., cancer, though the development of new sensitive tools remains an important challenge for researchers in this field. The sensitivity of the technique was sufficient to be able to determined data on the affinity of trastuzumab for the target receptor that were comparable with those previously reported, and the technique also provided access to kinetic data for this antibody-cell surface interaction, highlighting its potential for use in further studies of this and other cell-surface mediated interactions.

## 4. Conclusions/Outlook

In this work, we have studied the interaction between the antibody trastuzumab to SKOV3 cells, a human ovarian cancer cell line, fixed to COP-1 QCM sensor chips. We have also shown that the cell chip preparation (e.g., fixation procedure and seeding density) affects the cell conditions and binding response. This real time study, involving the direct measurement of interaction with intact cellular receptors provided quantitative binding data regarding both affinity and kinetics of interactions, in contrast with that generally derived from immunohistochemical assays. This approach offers potential for the screening of antibody derived HER2-directed candidate drugs and as a proof of concept for other binding studies involving membrane bound targets. These real time, label-free measurements, give sensitive and detailed kinetic information about the binding of the analyte to the cell-associated target as compared to label-dependent assays such as flow cytometry, fluorescence microscopy or standard microplate, *i.e.*, ELISA. Moreover, we have shown that this QCM cell biosensor can be used to provide information of biomolecular recognition processes in environments more like the natural environments of the target structures.
